# Characteristics of Homebound and Patients with Disability Who Use Home-Based Primary Care in Korea: A Retrospective Study for 2018–2022

**DOI:** 10.3390/jcm13061604

**Published:** 2024-03-11

**Authors:** Sun Young Lee, Hyein Chu, Yu-Mi Kim

**Affiliations:** 1 Public Healthcare Center, Seoul National University Hospital, Seoul 03080, Republic of Korea; sy2376@snu.ac.kr; 2 Department of Human System Medicine, Seoul National University College of Medicine, Seoul 03080, Republic of Korea; 3 Salim Clinic, Salim Health Welfare Social Cooperative, Seoul 03412, Republic of Korea; muyoung98@gmail.com; 4 Department of Preventive Medicine, Hanyang University College of Medicine, Seoul 04763, Republic of Korea; 5 Graduate School of Public Health, Hanyang University, Seoul 04763, Republic of Korea

**Keywords:** home-based primary care, homebound, disabled, home healthcare

## Abstract

(1) **Background and Methods**: This study evaluated characteristics of South Korean patients necessitating home-based primary care (HBPC) from 2018 to 2022, distinguishing between homebound individuals with chronic conditions and those with registered disabilities. (2) **Result**: Among 171 HBPC recipients, 56.1% were homebound, predominantly older with a median age of 81 years (interquartile range (IQR 68.5–86.0)), while 43.9% were disabled, generally younger with a median age of 39 years (IQR, 28–64). Activities of daily living were assessed, revealing a median score of 14 (IQR, 10–19), indicative of high care dependency. The most common conditions among homebound patients were dementia (27.1%) and physical mobility difficulties (21.9%), whereas mental disabilities (53.3%) and mobility issues (36.0%) prevailed in disabled patients. The primary HBPC needs for homebound patients included management of acute medical conditions (27.1%) and sores (17.7%). Conversely, regular health check-ups (46.7%) and management of neuropsychiatric symptoms (26.7%) were prevalent among the disabled group. (3) **Conclusion**: Notably, over 90% of HBPC patients required assistance with daily activities, highlighting significant differences in the needs and characteristics between older, homebound individuals with multiple comorbidities and younger, disabled patients receiving medical aid. These insights emphasize the necessity to develop customized HBPC programs to adequately cater to the diverse patient needs within South Korea.

## 1. Introduction

As the population ages, the number of homebound patients living with chronic diseases increases [1,2]. Homebound refers to a person who never or rarely leaves home on their own among the population aged 65 and older. A systematic review examining the prevalence of homebound populations revealed rates ranging from 5.6% to 19.6% in the United States, 9.8% to 39.8% in Spain, and 10.7% to 34.8% in Japan [3]. The estimated activity limitation among older adults in South Korea is 17–21%. As the number of patients who are unable to visit medical institutions independently increases, the importance of home-based medical care (HBMC) increases, as medical personnel visit patients’ homes to provide medical services [4,5].

Home-based primary care (HBPC) is a representative HBMC model that has proven effective in many countries [6,7]. Moreover, HBPC is a service in which a primary care provider visits the patient’s home and provides medical services when a patient with a chronic disease has difficulty moving [8]. In North American countries such as the US, HBPC was introduced in the early 1990s, and its effectiveness was verified in multiple randomized controlled trials [7,9]. Providing HBPC to homebound patients reduced acute care utilization, improved quality of life, and reduced medical costs [7,8,10]. Therefore, various attempts are being made to provide more HBPC to homebound patients [11,12].

HBPC is closely related to the primary care system of a country and applied differently depending on each country’s healthcare system [8,13]. However, limited data exist on HBPC programs outside North America, especially in Asia. In Japan, where aging is a serious concern, attempts are being made to provide HBPC by nurses [14]. South Korea also has a serious aging issue, with 18.4% of the population being 65 years or older in 2023 [15]. They have high accessibility to healthcare through the National Health Insurance Service (NHIS) [16]. However, HBPC is still in the introductory stage [17]. After hospital-based homecare nursing was introduced in 2001, HBPC with physician visits was first introduced in 2018 [17]. The HBPC service for the disabled began in 2018, and the more general service for homebound patients began in 2019 [18]. In both programs, a primary physician visits homebound patients living in the community to provide medical services. However, limited data exist on the types of patients using these programs in countries with high healthcare accessibility. Moreover, understanding the characteristics and medical needs of patients requiring HBPC services is necessary for the optimal design of HBMC services in an aging society, where the number of homebound patients is expectedly rising.

We investigated the characteristics and medical needs of Korean patients using HBPC and compared these characteristics according to the type of HBPC service.

## 2. Materials and Methods

### 2.1. Study Setting: HBMC in Korea and Study Clinic HBPC Team

Korea’s population is approximately 50 million. The Korean NHIS covers all types of healthcare services as a single insurer, such as inpatient, outpatient, and HBMC [16,17]. In 2001, hospital-based homecare nursing, in which a nurse visits the home of a patient discharged from the hospital, was initiated. HBPC with a physician visit was first introduced in 2018 [17]. The first HBPC service, launched in May 2018, was a pilot project for the primary care of people with disabilities. Physicians and nurses from the primary clinic completed 2 days of training and visited severely disabled people registered in the National Pension Service to provide medical services [18].

In 2019, a pilot project was implemented for primary care home visits for homebound patients, in which a primary care physician visits the home of a patient with mobility restrictions, regardless of whether the patient has a registered disability. This does not include home visits by nurses.

This study was conducted at a primary care clinic in Seoul with 12 physicians and 30,000 outpatient visits annually, as of 2022. This clinic opened as a health-welfare social cooperative in 2012. In 2018, one family physician participated in the HBPC for disabled people. In 2020, the clinic hired a dedicated visiting nurse. In 2021, another dedicated visiting nurse was hired and, along with one family physician, a psychiatry specialist began home visits. The clinic provides HBPC services to homebound patients who were previously treated at the clinic and patients from other clinics/hospitals who did not participate in HBPC programs.

### 2.2. Study Design and Data Source

This cross-sectional, observational study was conducted through a retrospective review of medical records. The records were registered by the physician, nurse, and social worker of the HBPC team during their first visit to the patient.

### 2.3. Study Population

Among the adult patients treated at the study clinic over 4 years from July 2018 to June 2022, patients enrolled in the HBPC service and visited by a physician were included in the study. Those who enrolled but had no visits by a physician were excluded.

### 2.4. Variables

The main factor of interest was the type of HBPC service. This includes patients registered as disabled or homebound. Some patients may be disabled and homebound; however, they are classified based on the registered program.

We reviewed the medical records registered by physicians, nurses, and social workers. During the first visit, more than two members of the HBPC team (a physician and a nurse or social worker) examined the patient at home. The variables used in the study were based on medical records investigated at the first home visit. Uninvestigated information at the first visit was collected during subsequent visits. The following patient information were investigated: (1) demographics (age, sex, type of health insurance (national health insurance and medical aid), long-term care insurance (LTCI, yes or no; if yes, grade 1 (totally dependent) to 4 (partially dependent), and 5 (dementia)), and disabled registration (yes or no; if yes, type of disability)); (2) decision maker and primary caregiver (self, spouse, parent, children, or social worker); (3) status at HBPC first visit (dependence in daily living (independent, partially dependent, or totally dependent), Korean version of activities of daily living (K-ADL, score 7–21) [19], communication (fluent, difficult, or unable), mental status (alert, drowsy, stupor, or coma), and global deterioration scale (GDS, score 1–7)) [20]; (4) chief medical condition (mental disability, dementia, cancer, stroke, fracture, frailty, physical mobility difficulty due to other reason, and others); and (5) medical condition at HBPC first visit (sore (yes or no), medical device (foley catheter, nasogastric tube, and tracheostomy), Charlson comorbidity score [21], Braden scale for predicting pressure sore risk (score 6–23) [22], and Huhn scale for fall risk (scale 0–28)) [23].

The following variables were investigated regarding the service provided by the HBPC team: (1) first visit year, (2) intake route (self, social welfare center, public community center, public health center, other clinic/hospital, and others), (3) primary reason for physician home visit (neuropsychiatric symptom management, regular health check-up, acute medical problem, sore management, rehabilitation, hydration, medical device management, post-operative care, nutritional consultation, and LTCI document), (4) HBPC team management (polypharmacy education, care resource coordination, and education for end-of-life care at home), (5) service coordination (social welfare center, public community center, public health center, other clinic/hospital, and long-term care institution—multiple choice), (6) reason for stopping visits, if applicable (institution admission (hospital and nursing home), death, problem solving, moving, and others), (7) period of HBPC service use, and (8) number of visits during use of the service (physician and nurse). As a physician’s opinion is required to use LTCI services in Korea, a visit for this purpose was classified as LTCI documentation of the purpose of the physician home visit [24]. Service coordination is the HBPC team connecting social resources after home visits. The reasons for ending the use of HBPC services during the study period were investigated. The period of service use was calculated from the time of registration to the end date of service or end date of study observation.

### 2.5. Statistical Analyses

Patient characteristics and medical services provided were compared according to the service type. Categorical variables are presented as counts and proportions. Continuous variables are presented as medians and interquartile ranges (IQRs). The numbers of visits by physicians and nurses per quarter are presented in a graph.

All statistical analyses were conducted using the SAS software (version 9.4; SAS Institute Inc., Cary, NC, USA).

### 2.6. Ethics Statement

The study was conducted according to the guidelines of the Declaration of Helsinki, the study protocol was approved by the institutional review boards of Hanyang University, and the need for informed consent was waived (protocol code HYUIRB-202205-022 issued on 20 May 2022).

## 3. Results

### 3.1. Study Population

Among the 11,376 adult patients treated at the study clinic over 4 years, 176 (1.55%) were registered with the HBPC program. Among these, 171 patients visited by physicians were included in the study.

### 3.2. Characteristics of HBPC Users by Type of Service

Of the 171 patients, 96 (56.1%) were homebound and 75 (43.9%) were disabled. The median (IQR) age of patients was 81 (68.5–86.0) years for homebound and 39 (28–64) years for disabled. Of these patients, 50.9% received medical aid (homebound 28.1% and disabled 80.0%) and 39.4% were registered as LTCI beneficiaries (homebound 51.0% and disabled 25.3%). For homebound patients, 58.3% of the decision makers were children and 26.0% were the patients themselves. Among disabled people, 44.0% were social workers and 36.0% were the patients themselves. Of the caregivers for the homebound, 51.0% were children and 20.8% were paid caregivers. For disabled patients, 84.0% were paid caregivers (Table 1).

### 3.3. Medical Condition of HBPC Users by Type of Service

More than 90% of patients were dependent in daily living (completely dependent, 42.7%; partially dependent, 49.7%). The overall K-ADL score (median, IQR) was 14 (10–19). The most common medical conditions of homebound patients were dementia (27.1%) and difficulty with physical mobility (21.9%). Among disabled patients, mental disability (53.3%) and difficulty in physical mobility (36.0%) were common. A CCI score of 3 or higher was observed in 64.6% of homebound patients (Table 2).

### 3.4. HBPC Team Home Visit Service by Type of Service

Although 60.4% of homebound people visited the HBPC clinic on their own, 81.3% of disabled people requested services from social welfare centers. For homebound patients, the most common primary reasons for physician home visits were acute medical problems (27.1%), neuropsychiatric symptom management (17.7%), and sore management (17.7%). Regular health check-ups (46.7%) and neuropsychiatric symptom management (26.7%) were also common. The HBPC team provided polypharmacy management in 39.8% (homebound 22.9% and disabled 61.3%) and care resource coordination in 39.8% of patients (homebound 42.7% and disabled 36.0%). During the study period, 75 patients (43.9%) stopped using the HBPC service. The common reasons were institutional admission (30.7%), death (28.0%), and resolution of the problem (26.7%) (Table 3).

### 3.5. Number of Home Visits and Period of HBPC Service Use by Type of Service

**Figure 1:**
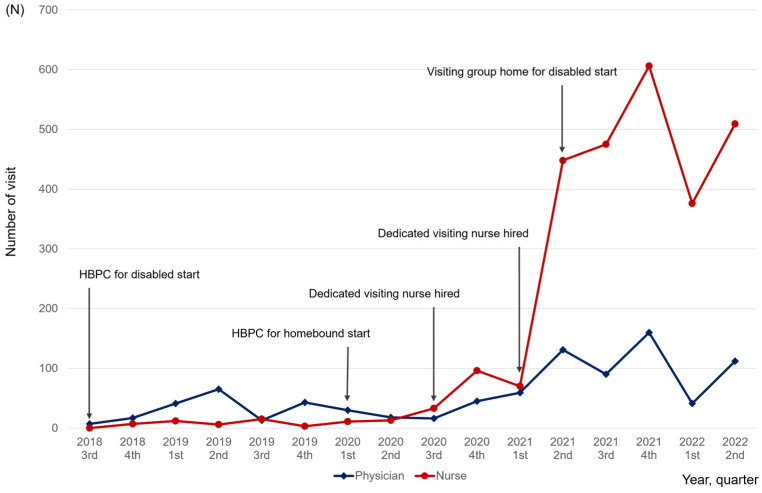
Quarterly number of visits by physician and nurse according to the program development.

From 2021, when a second dedicated visiting nurse was hired, the number of visits increased sharply (Figure 1).

The median (IQR) number of home visits per patient was 1 (1–2) for physicians and 5 (1–20) for nurses. For homebound patients, it was 1 (1–2) for physicians and 4 (1–12.5) for nurses. For disabled patients, it was 8 (4–11) for physicians and 9 (1–38) for nurses. The median (IQR) period of service use was longer for disabled (15.9 (10.7–41.0) months) than homebound (2.6 (0.7–7.6) months) patients. The median (IQR) visit number per patient per month was 0.4 (0.1–1.45) for physicians and 1.6 (0.5–4.6) for nurses for homebound, and 0.3 (0.2–1) for physicians and 1.6 (0–3.2) for nurses for disabled patients (Table 4).

## 4. Discussion

Less than 2% of patients treated at primary care clinics received HBPC. Of the patients who used HBPC services, 60% used HBPC for homebound programs and 40% for disabled programs. In both program groups, over 90% of patients utilizing HBPC demonstrated dependency in their daily activities. The users of the two HBPC programs exhibited distinct characteristics, needs, and patterns of usage. Disabled program users were younger and received more medical aid. Many homebound individuals had dementia or physical mobility difficulties and needed HBPC for acute problem-solving and sore management. Additionally, many disabled individuals had mental disabilities and physical mobility difficulties and require regular health check-ups and neuropsychiatric symptom management. During the study period, 40% of patients ended their use of the HBPC service, and the common reasons were institutional admission and death. Disabled individuals used HBPC services longer than those who were homebound.

This is the first study to investigate the characteristics of patients requiring HBPC in Korea. HBPC is an effective medical service in North American countries, such as the US and Canada [4,9,25]. Alternatively, in countries with universal health coverage and high healthcare accessibility, HBPC is in its introductory stage, and limited data exist regarding which patients use HBPC and its service characteristics. The strength of this study is that it thoroughly investigated the characteristics of patients and services provided at a primary care clinic that actively provided HBPC services from the beginning of the pilot project. Furthermore, we found that the patients enrolled in the two types of HBPC services had very different characteristics. A rough sketch shows that HBPC for disabled patients included many young patients receiving medical aid, with a median age of 40. For disabled individuals, care-related decisions were frequently made by social workers and they were cared for by paid caregivers. Conversely, the HBPC for homebound patients included many older adults in their 80s who had multiple comorbidities. Children mainly played the roles of decision makers and caregivers. Based on these differences in characteristics, the HBPC services required by the two groups also differed. Homebound patients required HBPC services to solve acute medical problems and manage their sores. However, disabled patients were younger and often required periodic visits for ongoing health check-ups and medication adjustments for neuropsychiatric symptoms. Therefore, patients registered in the disabled service used HBPC services longer than homebound patients. Mobility problems or acute medical problems can be resolved for the homebound; nevertheless, regular health check-ups are necessary for people with disabilities whose mobility problems do not improve. Moreover, most of the patients who used hospital-based homecare nursing services in a tertiary hospital in Korea had serious conditions such as cancer and degenerative neurologic disorders [17]. They needed support for essential functions such as respiratory function, nutrition support, and management of medical devices [17]. Nevertheless, in our study, which analyzed patients using HBPC services provided by community primary care clinics, few patients were dependent for essential functions of life or needed medical device management. Patients requiring HBPC require medication adjustment for chronic conditions and management of acute medical conditions, which are provided in primary care clinics [26,27]. Thus, homebound patients and people with disabilities still need primary care at home.

The two groups were dependent in their daily lives, and about half of them needed care coordination. The HBPC service is characterized by a physician visiting the patient’s home instead of the patient visiting the clinic. Therefore, patients with mobility difficulties are the primary targets. Furthermore, HBPC for disabled did not necessarily target only people with physical mobility issues; depending on the type of disability, many patients would have been “homebound” [28,29]. As these patients have difficulty accessing medical care, HBPC may be helpful.

Among the patients who ended their use of HBPC, only one-quarter stopped it because the problem was resolved. Most of the patients were admitted to institutions or died. The results showed that many patients requiring HBPC experienced deteriorating mobility and struggled to stay at home. As the number of older adults increases, the number of patients requiring HBPC services continues to increase. Thus, preparation for the expansion of services is crucial. Additionally, 30% of the patients terminated the service due to death. These patients died at home or stayed at home until just before death and then died in a hospital. In Korea, many people hope to die at home; however, the actual number of patients who die at home is small [30]. This may be attributed to the absence of home-based services to support end-of-life care, education, and symptom control [31]. There is a potential to support home death through HBPC [32]

The number of home visits per patient by nurses was higher than that by physicians in both services. The HBPC for disabled individuals includes a nurse visit. However, the HBPC for homebound individuals does not include official nurse visits. Nevertheless, the outcomes of the service showed that it is difficult to operate with only a physician visit and that a nurse visit is also necessary for HBPC for homebound patients. In Japan, nurses provide HBPC services at nursing stations [14] and in the US HBPC model, nurse’s role is pivotal [9,33]. It is difficult to apply the model of the US to Korea, which does not have nurse practitioners [9]. Additionally, it is necessary to develop an optimal model that fits the Korean situation.

In Korea, where the NHI and LTCI are universally applied, it is common for homebound patients to be institutionalized in long-term care hospitals or stay in nursing homes [24]. However, some patients prefer to stay at home even after their mobility becomes impaired [34]. For these patients, HBPC is the only way to receive necessary medical services. In Korea, which is an aging society, the need for HBPC is expected to increase [17,35]. It is time to think about how to provide optimal HBPC to suit the characteristics and needs of homebound individuals and patients with disabilities. This study provides the basic data for designing tailored HBPC services.

This study had several limitations. First, as this was a single-center study, it is difficult to generalize the results to HBPC programs conducted throughout Korea. However, in a situation where HBPC is not provided as a universal service, the experience of clinics actively providing services on a community basis provides valuable information for service advancement. Second, patient characteristics were examined based on their status and needs at the time of the first home visit. Patients using HBPC for a long time may have more diverse service needs than those investigated in this study. The results of this study can be viewed as a primary requirement for patients who require HBPC.

## 5. Conclusions

More than 90% of the patients who require HBPC are dependent on it for their daily activities. The characteristics of patients and their medical needs were different for older homebound patients with multiple comorbidities and young disabled patients under medical aid. It is time to consider providing optimal HBPC tailored to the characteristics and needs of patients with limited mobility who wish to stay at home.

## Figures and Tables

**Table 1:** Patient characteristics of home-based primary care patients by type of service.

		Total		Homebound		Disabled	
		N	%	N	%	N	%
Total		171	100.0	96	100.0	75	100.0
Sex	Female	74	43.3	53	55.2	21	28.0
Age, year							
	19~44	48	28.1	4	4.2	44	58.7
	45~64	23	13.5	10	10.4	13	17.3
	65~79	46	26.9	32	33.3	14	18.7
	80~	54	31.6	50	52.1	4	5.3
	Median (IQR)	69 (41–83)		81 (68.5–86)		39 (28–64)	
Health insurance							
	NHI	84	49.1	69	71.9	15	20.0
	Medical aid	87	50.9	27	28.1	60	80.0
LTCI							
	No	103	60.2	47	49.0	56	74.7
	Yes	68	39.8	49	51.0	19	25.3
	LTCI grade (N = 68)						
	1	16	23.5	7	14.3	9	47.4
	2	18	26.5	12	24.5	6	31.6
	3	22	32.4	18	36.7	4	21.1
	4	11	16.2	11	22.4	0	0.0
	5	1	1.5	1	2.0	0	0.0
Disabled registration							
	No	74	43.3	74	77.1		
	Yes	97	56.7	22	22.9	75	100.0
	Type of disabled (N = 97)						
	Developmental	38	39.2	1	4.5	37	49.3
	Brain injury	35	36.1	10	45.5	25	33.3
	Physical	16	16.5	8	36.4	8	10.7
	Others ^1^	8	8.2	3	13.6	5	6.7
Living at group home for disabled		27	15.8	-	-	27	36.0
Decision maker							
	Patient self	52	30.4	25	26.0	27	36.0
	Spouse	13	7.6	9	9.4	4	5.3
	Parent	11	6.4	5	5.2	6	8.0
	Children	61	35.7	56	58.3	5	6.7
	Social Worker	34	19.9	1	1.0	33	44.0
Caregiver							
	Patient self	7	4.1	7	7.3	0	0.0
	Spouse	19	11.1	16	16.7	3	4.0
	Parent	9	5.3	4	4.2	5	6.7
	Children	53	31.0	49	51.0	4	5.3
	Paid caregiver	83	48.5	20	20.8	63	84.0

N, number; IQR, interquartile range; NHI, national health insurance; LTCI, long-term care insurance. ^1^ Others include visual and hearing disabilities.

**Table 2:** Medical condition of home-based primary care patients by type of service.

		Total		Homebound		Disabled	
		N	%	N	%	N	%
Total		171	100.0	96	100.0	75	100.0
Daily living							
	Independent	13	7.6	9	9.4	4	5.3
	Partially dependent	85	49.7	44	45.8	41	54.7
	Totally dependent	73	42.7	43	44.8	30	40.0
K-ADL	Median (IQR)	14 (10–19)		14 (11–19)		13 (8–18)	
Communication							
	Fluent	84	49.1	56	58.3	28	37.3
	Difficult	65	38.0	33	34.4	32	42.7
	Unable	22	12.9	7	7.3	15	20.0
Alertness							
	Alert	144	84.2	76	79.2	68	90.7
	Drowsy	16	9.4	13	13.5	3	4.0
	Stupor	9	5.3	7	7.3	2	2.7
	Coma	2	1.2	0	0.0	2	2.7
GDS (N = 116)							
	1, normal	31	31.6	23	28.8	8	44.4
	2	17	17.3	15	18.8	2	11.1
	3	18	18.4	17	21.3	1	5.6
	4	10	10.2	9	11.3	1	5.6
	5	13	13.3	10	12.5	3	16.7
	6	15	15.3	11	13.8	4	22.2
	7, severe	12	12.2	9	11.3	3	16.7
	Median (IQR)	3 (1–5)		3 (2–5)		3.5 (1–6)	
	Uncheckable	55	56.1	2	2.0	53	54.1
Chief medical condition							
	Physical mobility difficulty	48	28.1	21	21.9	27	36.0
	Mental disability	44	25.7	4	4.2	40	53.3
	Dementia	27	15.8	26	27.1	1	1.3
	Cancer	19	11.1	17	17.7	2	2.7
	Stroke	15	8.8	10	10.4	5	6.7
	Fracture	8	4.7	8	8.3	0	0.0
	Frailty	4	2.3	4	4.2	0	0.0
	Others	6	3.5	6	6.3	0	0.0
Sore		36	21.1	25	26.0	11	14.7
Medical device							
	Foley catheter	21	12.3	13	13.5	8	10.7
	Nasogastric tube	5	2.9	2	2.1	3	4.0
	Tracheostomy	3	1.8	1	1.0	2	2.7
CCI							
	0	59	34.5	11	11.5	48	64.0
	1	24	14.0	13	13.5	11	14.7
	2	13	7.6	10	10.4	3	4.0
	3+	75	43.9	62	64.6	13	17.3
Braden scale for pressure sore risk							
	19–23, no risk	79	61.2	31	33.0	48	137.1
	15–18, little risk	38	29.5	30	31.9	8	22.9
	13–14, moderate risk	28	21.7	20	21.3	8	22.9
	10–12, high risk	14	10.9	7	7.4	7	20.0
	~9, very high risk	12	9.3	8	8.5	4	11.4
	Median (IQR)	18 (14–23)		16 (13.5–19.5)		22 (14–23)	
Huhn scale for fall risk (N = 109)				N = 85		N = 24	
	~4, low risk	2	1.5	2	2.2	0	0.0
	5–10, moderate risk	20	14.6	18	19.4	2	4.5
	11~, high risk	87	63.5	65	69.9	22	50.0
	Median (IQR)	16 (11–19)		15 (11–19)		17 (15–20)	
	Non-indicated (<60 yr)	62	48.1	11	8.5	51	39.5

N, number; K-ADL, Korean activities of daily living; GDS, global deterioration scale; CCI, Charlson comorbidity index.

**Table 3:** Home visit service by home-based primary care team by type of service.

		Total		Homebound		Disabled	
		N	%	N	%	N	%
Total		171	100.0	96	100.0	75	100.0
First visit year							
	2018	17	9.9			17	22.7
	2019	13	7.6			13	17.3
	2020	20	11.7	11	11.5	9	12.0
	2021	88	51.5	62	64.6	26	34.7
	2022	33	19.3	23	24.0	10	13.3
Intake route							
	Self	65	38.0	58	60.4	7	9.3
	Social welfare center	65	38.0	4	4.2	61	81.3
	Public community center	24	14.0	23	24.0	1	1.3
	Public health center	8	4.7	3	3.1	5	6.7
	Other clinic/hospital	3	1.8	2	2.1	1	1.3
	Others	6	3.5	6	6.3	0	0.0
Primary physician visit reason							
	Neuropsychiatric symptom management	37	21.6	17	17.7	20	26.7
	Regular health check-up	35	20.5	0	0.0	35	46.7
	Acute medical problem ^1^	33	19.3	26	27.1	7	9.3
	Sore management	25	14.6	17	17.7	8	10.7
	Rehabilitation	13	7.6	13	13.5	0	0.0
	Hydration	12	7.0	12	12.5	0	0.0
	Medical device management	9	5.3	5	5.2	4	5.3
	Post-operative care	3	1.8	3	3.1	0	0.0
	Nutritional consultation	2	1.2	1	1.0	1	1.3
	LTCI document	2	1.2	2	2.1	0	0.0
Home healthcare team management							
	Polypharmacy education	68	39.8	22	22.9	46	61.3
	Care resource coordination	68	39.8	41	42.7	27	36.0
	Home death education	26	15.2	24	25.0	2	2.7
Service coordination (multiple choice)							
	Public health center	48	28.1	13	13.5	35	46.7
	Other clinic/hospital	30	17.5	23	24.0	7	9.3
	Social welfare center	21	12.3	1	1.0	20	26.7
	Public community center	12	7.0	11	11.5	1	1.3
	Long term care institution	8	4.7	7	7.3	1	1.3
Visit end reason (N = 75)							
	Institution admission	23	30.7	19	30.6	4	30.8
	Death	21	28.0	19	30.6	2	15.4
	Problem solving	20	26.7	19	30.6	1	7.7
	Moving	7	9.3	4	6.5	3	23.1
	Others ^2^	4	5.3	1	1.6	3	23.1

N, number; LTCI, long-term care insurance. ^1^ Acute medical problem including fever, dehydration, and so on. ^2^ Others include relationship with team and cost.

**Table 4:** Number of visits and period of home-based primary care service by type of service.

Variable		Total	Homebound	Disabled
Patient number, N		171	96	75
Visit number, median (IQR)				
	Physician	2 (1–7)	1 (1–2)	8 (4–11)
	Nurse	5 (1–20)	4 (1–12.5)	9 (1–38)
Period of service use, median (IQR)				
	day	220 (68–476)	78.5 (21.5–228)	476 (322–1231)
	month	7.3 (2.3–15.9)	2.6 (0.7–7.6)	15.9 (10.7–41.0)
Visit number per month, median (IQR)				
	Physician	0.4 (0.2–1.2)	0.4 (0.1–1.45)	0.3 (0.2–1)
	Nurse	1.6 (0.1–3.8)	1.6 (0.5–4.6)	1.6 (0–3.2)

N, number; IQR, interquartile range.

## Data Availability

The data used came from Salim clinic, and restrictions apply to the availability of these data. However, the data might be available from the corresponding author upon reasonable request and with permission of the Salim Health Welfare Social Cooperative.
